# The effect of intranasal oxytocin versus placebo treatment on the autonomic responses to human sounds in autism: a single-blind, randomized, placebo-controlled, crossover design study

**DOI:** 10.1186/2040-2392-5-20

**Published:** 2014-02-28

**Authors:** I-Fan Lin, Makio Kashino, Haruhisa Ohta, Takashi Yamada, Masayuki Tani, Hiromi Watanabe, Chieko Kanai, Taisei Ohno, Yuko Takayama, Akira Iwanami, Nobumasa Kato

**Affiliations:** 1 NTT Communication Science Laboratories, NTT Corporation, 3-1, Morinosato Wakamiya, Atsugi, Kanagawa 243-0198, Japan; 2 CREST, JST, 3-1, Morinosato Wakamiya, Atsugi, Kanagawa 243-0198, Japan; 3 Department of Psychiatry, Showa University School of Medicine, 1-5-8 Hatanodai, Shinagawa, Tokyo 142-8555, Japan; 4 CREST, JST, 1-5-8 Hatanodai, Shinagawa, Tokyo 142-8555, Japan

**Keywords:** Autism, Oxytocin, Clinical trial, Auditory, Social cognition, Skin conductance response, Autonomic system

## Abstract

**Background:**

Many individuals with autism spectrum disorders (ASD) have difficulty with verbal communication, which might be due to a lack of spontaneous orientation toward social auditory stimuli. Previous studies have shown that a single dose of oxytocin improves speech comprehension in autism. The primary aim of this study was to investigate whether the orientation behaviors toward human sounds are different for neurotypical (NT) adults and adults with ASD and whether oxytocin has an effect on their orientation behaviors toward human sounds.

**Methods:**

This was a randomized, placebo-controlled, within-subject, crossover design study of intranasal oxytocin versus placebo in 13 NT adults and 16 adults with ASD. Subjects were randomized to 24 IU intranasal oxytocin or placebo on different days, and they were blind to the treatment. The participants then listened passively to human and non-human affective sounds while their skin conductance responses (SCRs) and the changes in peripheral blood vessel constriction were monitored as an indicator of spontaneous orientation. The monitored data were analyzed by a mixed-design ANOVA.

**Results:**

Oxytocin enhanced the difference between the SCRs to human and non-human sounds in both the NT and ASD groups (F(1,56) = 6.046, *p* = 0.017). Further correlation coefficient analysis showed significant correlations between this SCR difference and the scores in the autism spectrum quotient ‘attention to detail’ and ‘social skill’ subscales and interpersonal reactivity index and social functioning scale in the ASD group. Oxytocin was well tolerated, and no serious adverse effects were reported.

**Conclusions:**

The difference in SCRs implies that oxytocin nasal spray may enhance orientation behaviors toward human sounds in the presence of other environmental sounds in both ASD and NT adults.

**Trial registration:**

UMIN-CTR Clinical Trial, Unique trial number: UMIN000005809

## Background

Social communication is a highly valued skill, and those who have difficulty in interacting with others may find it hard to become fully integrated into society. Many individuals diagnosed as having autism spectrum disorders (ASD) are impaired in terms of social skills and the social use of verbal and non-verbal communication. Although individuals with Asperger syndrome (AS) are usually adept at the linguistic aspect of communication, they still have difficulty with the paralinguistic component. In other words, they may have normal verbal ability, but their speech may exhibit unusual prosody, volume, or intonation. Some researchers have proposed that the early emergence of a lack of spontaneous orientation toward social stimuli in individuals with ASD disturbs the emergence of social and communication skills [1]. For example, in the vision domain, individuals with ASD are found to exhibit abnormal visual fixation patterns for the eye regions [2-5] and abnormal brain activity patterns for face processing [6,7]. In the auditory domain, individuals with ASD are found to pay less attention to speech sounds [8] and show abnormal brain activity patterns for voice processing [9,10]. This lack of attention and response to social stimuli may delay the development of verbal and non-verbal communication skills, which are strong predictors of later outcomes in individuals with ASD [11].

While the pathogenesis of autism is unclear and the treatment for autism is limited, an interesting current hypothesis has been proposed that oxytocin might alleviate the social disorders observed as the hallmark of ASD. Previous studies have found the association of the oxytocin receptor (OXYR) gene polymorphisms with autism in Chinese Han, Caucasians, and Japanese individuals [12-14]. These genetic variations of OXYR are found to cause morphometric alterations of the hypothalamus, anterior cingulate cortex, and amygdala [15-18]. After oxytocin administration, the brain activation patterns in the fusiform gyrus are found to increase in the risk allele carriers [19]. This pharmaco-imaging genetics study provides a possible mechanism for oxytocin to alleviate the social disorders in ASD.

In the past decade, oxytocin has been found to play an important role in social interaction that goes beyond its previously known effects on female reproduction [20]. Because a very significant component of ASD is related to social interaction, some pioneering researchers have investigated the effect of oxytocin on individuals with ASD (Table 1). Compared with those receiving a placebo, individuals with ASD receiving the oxytocin treatment exhibit fewer repetitive behaviors [21] and improved social interaction [22], eye gaze behaviors [22], performance in the reading the mind in the eyes test [23,24], and affective speech perception [25]. In addition, oxytocin increases brain activity patterns in the right amygdala in individuals with ASD during facial processing, and this finding supports the view that oxytocin increases the salience of facial stimuli in the ASD group [26].

**Table 1:** Summary of the findings in previous studies that investigated the effect of oxytocin in ASD

**Study**	**Oxytocin application**	**Major findings**
Hollandar et al. (2003) [21]	Intravanous	Reduced repetitive behaviors
Hollandar et al. (2007) [25]	Intravanous	Improved affective speech comprehension
Guastella et al. (2010) [23]	Intranasal (single dose)	Improved performance in Reading-the-Mind-in-the-Eyes Test
Andari et al. (2010) [22]	Intranasal (single dose)	Improved social behaviors, subjective feeling of trust, gazing behaviors at the eye region
Anagnostou et al. (2012) [24]	Intranasal (6 weeks)	No change in Diagnostic Analysis of Nonverbal Accuracy & Repetitive Behavior Scale Revised; improved performance in Reading-the-Mind-in-the-Eyes Test and quality of life
Domes et al. (2013) [26]	Intranasal (single dose)	Increased brain activity patterns in the right amygdala during face processing

While communication difficulty is one of the major symptoms observed in individuals with ASD, only a few studies have focused on the effect of oxytocin on their auditory perception. In light of the above findings, this randomized, single-blind, placebo-controlled, crossover design study was aimed at investigating whether oxytocin influences the orientation toward human sounds in ASD and NT individuals. In the experiment, when the participants listened passively to different sounds, we monitored their skin conductance responses (SCRs) and the changes in peripheral blood vessel constriction (by using photoplethysmography, PPG) as an indicator of their vigilance and emotional responses [27,28]. If oxytocin increased autonomic responses when individuals with ASD listen to human sounds, the results would indicate the potential of oxytocin to improve social communication skills in those individuals by enhancing their orientation toward human sounds.

## Methods

### Participants

Twenty-two male adults with ASD and 18 NT male adults participated in this experiment. The reason for taking only one sex was to avoid an additional source of noise in the effect of oxytocin across genders. Some participants’ physiology data were not completely recorded, so only the complete data from 16 participants with ASD (mean age = 32.56, range = 19-51) and 13 NT participants (mean = 34.69, range = 23-45) were included in the analysis.

The diagnosis of ASD was based on the consensus of three experienced psychiatrists and one psychologist according to the criteria of Diagnostic and Statistical Manual of Mental Disorders (DSM-IV-TR), fourth edition [29], after two detailed interviews conducted independently by a psychiatrist and a clinical psychologist in the team at Karasuyama Hospital, including their developmental history, present illness, past history, and family history [30]. Of the 16 ASD participants, 6 were diagnosed as having Asperger’s syndrome (AS), 8 were diagnosed as having high-functioning autism (HFA), and 2 were diagnosed as having pervasive developmental disorder not otherwise specified (PDD-NOS). None of the participants met the DSM-IV-TR criteria for any other psychiatric disorders.

Fourteen of the 16 participants with ASD received a WAIS-R IQ test (mean = 102.86, SD = 17.03). All the participants with ASD were evaluated regarding their autistic traits and social skills, including autism spectrum quotient (AQ) (mean = 36.88, SD = 5.18), interpersonal reactivity index (IRI) (mean = 71.06, SD = 8.50), and social functioning scale (SFS) (mean = 106.13, SD = 20.10). Fourteen of the 16 participants with ASD and 12 of the 13 NT participants received the Japanese version of the national adult reading test to estimate their IQ [31] (mean ± SD ASD group: 108.83 ± 11.68; NT group: 110.87 ± 10.21). There was no significant difference between the ages and IQ scores of these two groups.

The participants with ASD were recruited from the clinics without payment, and the NT participants were paid for their participation. Three of the 16 participants with ASD were taking medicine constantly (including selective serotonin reuptake inhibitors, benzodiazepine, or serotonin and norepinephrine reuptake inhibitors) during the experiment. All participants refrained from taking caffeine and alcohol on the test day. All procedures were in accordance with the Declaration of Helsinki and were approved by the Ethics Committees of the Faculty of Medicine of Showa University.

### Procedure

All the participants gave written informed consent before participation. In the randomized, placebo-controlled, crossover designed trial, two experimental sessions were conducted, separated by a 1-week interval. Each experiment started with a screening of current somatic illness. Oxytocin and placebo were administered intranasally 1 h before the auditory experiment [32]. After a standardized protocol, participants self-administered three puffs of oxytocin per nostril (Syntocinon-Spray, Novartis, Basel, Switzerland; each puff with 4 IU oxytocin; 24 IU oxytocin in total) or placebo under the supervision of a physician. Due to the regulations of the hospital, this attending physician was required to be aware of the medication that was handed to the participant. Nevertheless, this physician did not look into the data after it was collected, nor was he involved in data analysis and interpretation. Eight of the 16 ASD participants and 7 of the 13 NT participants inhaled oxytocin in their first session.

During the experiment, skin conductance sensors were fixed on the volar side of the left middle and ring fingers, and the PPG sensor was fixed on the volar side of the left index finger. The analog signals from these sensors were sampled at 1,000 Hz and then amplified and filtered with a BIOPAC MP150 (BIOPAC Systems Inc., USA). The digital signals from the skin conductance sensors (TSD203, BIOPAC Systems Inc., USA) were processed with a 1 Hz low-pass filter, and the digital signals from the PPG sensor (TSD200, BIOPAC Systems Inc., USA) were processed with a 0.5-3 Hz band-pass filter. The usage of these filters is recommended by BIOPAC System, Inc. The participants were prepared and then seated in a dark room with their left arm placed in a comfortable position. They were told not to move their left hand through the experiment. The experiment lasted for about 20 min.

### Auditory stimuli

**Figure 1:**
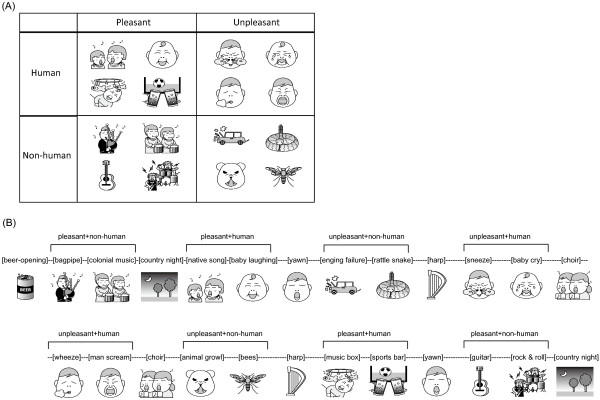
Illustrations for (A) emotional sounds in the four categories and (B) an example of the auditory sequence presented in the experiment.

All the auditory stimuli were chosen from International Affective Digital Sounds (IADS-2). There were four categories of sounds tested in the experiment (illustrated in Figure 1A): pleasant and human, pleasant and non-human, unpleasant and human, and unpleasant and non-human. These sounds were categorized as pleasant and unpleasant sounds based on their emotion evaluation [33]. Human sounds were sounds generated by human bodies, and non-human sounds included animal sounds, environmental sounds, and sounds generated by musical instruments. The categorization of human and non-human sounds was based on previous studies that investigated the brain activity patterns and response time evoked by human and non-human sounds [34,35]. There were no significant differences in the arousal level among these four categories of sounds (see Additional file 1: Table S1). In addition to these four categories, there was a sound of opening a beer can (pleasant and high-arousal) and four pleasant low-arousal sounds.An example of the sound sequence is illustrated in Figure 1B. The two sounds in the same category and listed in the same row in Figure 1A were always presented as a set. Every set of the sounds was followed by a pleasant low-arousal sound so that the participants had a chance to switch their mood. The sound of opening a beer can served as the first sound. The order of these sets was pseudo-randomized based on two rules to avoid the effect of expectations and the order effect. Initially, the first set in one category was presented in the first half of the experiment, and the second set was presented in the second half. Then, the two sets in the same category were arranged in a complementary manner. For example, in one experimental session, if the first set in the pleasant human category was presented first among the four sets in the first half session, the second set in the same category would be presented last among the four sets in the second half session.

In total there were 25 sounds presented in the experiment. Each sound was 6 s long, and the inter-stimulus intervals between these sounds were randomized and between 6 and 24 s. The first sound was presented after the participants had rested for 3 min. All the auditory files selected from the IADS-2 database were re-sampled to 44,100 Hz and stored in iPod model #A1199 (Apple, USA). The output sound pressure level was controlled by a digital linked headphone amplifier, Audio-Technica AT-HA35i (Audio-Technica Corp., Japan). The sound pressure levels were varied to match the sound pressure levels of sounds in a natural environment. The maximum sound pressure level was 76 dBSPL across all auditory stimuli. The auditory stimuli were presented to the participants diotically through a set of headphones (Sennheiser HDA 200, Germany).

### Data analysis

Due to the fast presentation of auditory stimuli, the 1-s pre-sound skin conductance level (SCL) was averaged to give a baseline for each auditory stimulus. To investigate whether the alertness of participants decreased over time due to adaptation or tiredness, the baseline SCLs for all auditory stimuli (shown in Additional file 1: Figure S1) except the first sound for all participants were analyzed by a two-way repeated-measurement ANOVA, with the within-subject factors as *Medication* and *Order*. Neither *Medication* nor *Order* showed a significant effect (F(1,28) = 0.021, *p* = 0.887 and F(23,644) = 0.315, *p* = 0.821, respectively). The interaction between *Medication* and *Order* was not significant either (F(23,644) = 0.363, *p* = 0.849). The results indicate that participants’ alertness toward the auditory stimuli was stable across time. On the other hand, because the range of the participants’ age was large, the Pearson correlation coefficients between participants’ age and their baseline SCLs for the first sound (of opening a beer can) for all participants in the placebo and oxytocin sessions was computed to investigate whether elder participants had lower baseline SCLs. No significant correlation was found (*p* = 0.47 in the placebo sessions and *p* = 0.2 in the oxytocin sessions). The lack of significant correlation between age and baseline SCLs in the present study is consistent with the previous study [36].

To find the stimulus-related peak of SCLs (i.e., SCRs), this baseline was subtracted from the maximum SCL between 1 s after the onset of each sound and 5 s after the offset of each auditory stimulus, and the residual went through a log transform (log [SCR + 1]) for normalization before further statistical analysis. The procedures for analyzing the skin conductance data and PPG data were determined before the experiment based on a previous study [37].

For the PPG signals, the peaks and valleys in the waveforms were detected throughout the signals for amplitude computation in the beginning. Then the 2-s pre-sound PPG amplitude was averaged to serve as the baseline. The minimum PPG amplitude between 1 s after the onset of each sound and 5 s after the offset of each sound was subtracted from the baseline before further statistical analysis.

## Results

The SCRs and PPG amplitude changes in each of the four sound categories (pleasant human, pleasant non-human, unpleasant human, unpleasant non-human) were averaged for each participant in each session. To investigate whether oxytocin influenced how subjects reacted to human and non-human sounds, whether the pleasantness of the sounds affected the effect of oxytocin, and whether the effect of oxytocin was the same for the subjects in the ASD and NT groups, a five-way mixed-design ANOVAs, with the between-subject factor as *Group* (ASD and NT) and *Measure* (SCR and PPG) and the within-subject factors as *Medication* (oxytocin and placebo), *Source* (human and non-human sound), and *Pleasantness* (pleasant and unpleasant sound), was performed for the averaged SCRs and PPG amplitude changes. The ANOVA analysis was conducted with SPSS v.19 (IBM, USA).

The results of the ANOVA, which are summarized in Table 2, show a significant effect of *Measure* and significant interactions between *Group* and *Measure*, *Medication* and *Group*, *Medication* and *Group* and *Measure*, *Medication* and *Source*, and *Medication* and *Source* and *Measure*. Autonomic responses (SCRs and PPG amplitude changes) were significantly larger in the NT group than in the ASD group. The subgroup analyses exploring the significant interaction between *Group* and *Measure* indicated that SCRs were larger in the NT group than in the ASD group, while PPG amplitude changes were larger in the ASD group than in the NT group, although none of these differences was significant.

**Table 2:** **The results of the five-way mixed-design repeated-measurement ANOVA, with the between-subject factor as *Group *** **(ASD and NT) and *Measure *** **(SCR and PPG) and the within-subject factors as *Medication *** **(oxytocin and placebo), *Source *** **(human and non-human sound), and *Pleasantness *** **(pleasant and unpleasant sound), performed to evaluate the effect of oxytocin inhalation on the autonomic responses**

	**df**	**F**	***p***
Group	1, 112	2.349	0.128
Measure	1, 112	18.718	0.000***
Group *Measure	1, 112	4.177	0.043*
Medication	1, 112	0.790	0.376
Medication *Group	1, 112	7.000	0.009**
Medication *Measure	1, 112	1.553	0.215
Medication *Group *Measure	1, 112	7.080	0.009**
Source	1, 112	0.458	0.500
Source *Group	1, 112	0.951	0.332
Source *Measure	1, 112	0.144	0.705
Source *Group *Measure	1, 112	0.701	0.404
Pleasantness	1, 112	2.194	0.141
Pleasantness *Group	1, 112	0.133	0.716
Pleasantness *Measure	1, 112	0.180	0.672
Pleasantness *Group *Measure	1, 112	0.296	0.587
Medication *Source	1, 112	4.384	0.039*
Medication × Source *Group	1, 112	1.976	0.163
Medication × Source *Measure	1, 112	5.802	0.018*
Medication × Source *Group *Measure	1, 112	1.373	0.244
Medication × Pleasantness	1, 112	0.008	0.929
Medication × Pleasantness *Group	1, 112	0.007	0.935
Medication × Pleasantness *Measure	1, 112	0.051	0.822
Medication × Pleasantness *Group *Measure	1, 112	0.112	0.739
Source × Pleasantness	1, 112	1.103	0.296
Source × Pleasantness *Group	1, 112	0.081	0.777
Source × Pleasantness *Measure	1, 112	0.058	0.810
Source × Pleasantness *Group *Measure	1, 112	0.141	0.708
Medication × Source × Pleasantness	1, 112	0.033	0.855
Medication × Source × Pleasantness *Group	1, 112	0.966	0.328
Medication × Source × Pleasantness *Measure	1, 112	0.134	0.715
Medication × Source × Pleasantness *Group *Measure	1, 112	0.918	0.340

*p < 0.05 **p < 0.01 ***p < 0.001.

**Figure 2:**
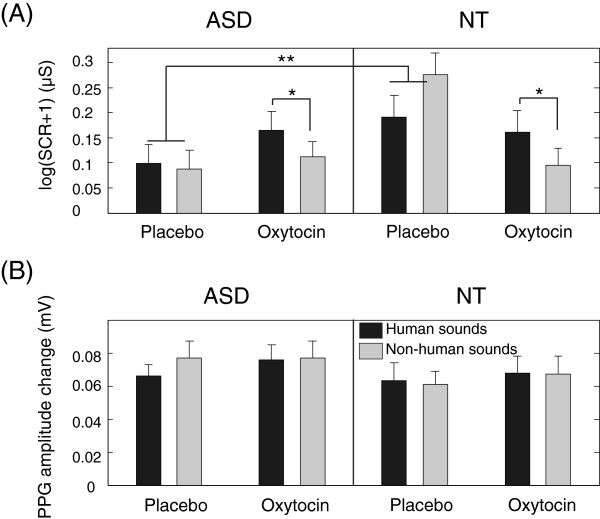
**The effect of oxytocin/placebo inhalation on (A) the skin conductance responses and (B) the PPG amplitude changes in response to human and non-human sounds in the ASD and NT groups.** The values shown are means over participants, and the *error bars* indicate standard errors.

The subgroup analyses exploring the significant interaction between *Medication* and *Group* and *Measure* showed that the interaction between *Medication* and *Group* was mainly due to the significant interaction between *Medication* and *Group* in SCR measurement (F(1,56) = 7.526, *p* = 0.008) but not in PPG measurement (F(1,56) = 0.001, *p* = 0.976). Further subgroup analyses showed the significant interaction between *Medication* and *Group* in SCR measurement was mainly due to the difference between the ASD and NT groups when they inhaled placebo (F(1,56) = 10.069, *p* = 0.002) and the significant difference between oxytocin and placebo inhalation in the NT group (F(1,25) = 4.991, *p* = 0.035). In Figure 2A, SCRs were larger in the NT group than in the ASD group after placebo inhalation, and this difference disappeared after oxytocin inhalation.

The subgroup analyses exploring the significant interaction between *Medication* and *Source* and *Measure* showed that the interaction between *Medication* and *Source* was mainly due to the significant interaction between *Medication* and *Source* in SCR measurement (F(1,56) = 5.226, *p* = 0.026) but not in PPG measurement (F(1,56) = 0.816, *p* = 0.37). Further subgroup analyses showed the significant interaction between *Medication* and *Source* in SCR measurement was mainly due to the significant difference for human and non-human sounds after oxytocin inhalation (F(1,56) = 6.046, *p* = 0.017). In Figure 2A, SCRs were larger for human sounds than for non-human sounds after oxytocin inhalation.

Although there was no significant interaction between *Medication*, *Source*, and *Group* in SCRs, the experimental hypothesis predicts that oxytocin should have different effects on the SCRs in the NT and ASD groups. Therefore, four two-way repeated-measurement ANOVAs (with the within-subject factors as *Sociality* and *Pleasantness*) were conducted to analyze the SCRs in the oxytocin and placebo conditions in the ASD and NT groups separately. For the NT participants, *Sociality* was not a significant factor in the placebo session (F(1,25) = 1.242, *p* = 0.276) or in the oxytocin session (F(1,25) = 2.172, *p* = 0.153). For the ASD participants, *Sociality* was not a significant factor in the placebo session (F(1,31) = 0.339, *p* = 0.565) but was a significant factor in the oxytocin session (F(1,31) = 4.913, *p* = 0.034).

**Figure 3:**
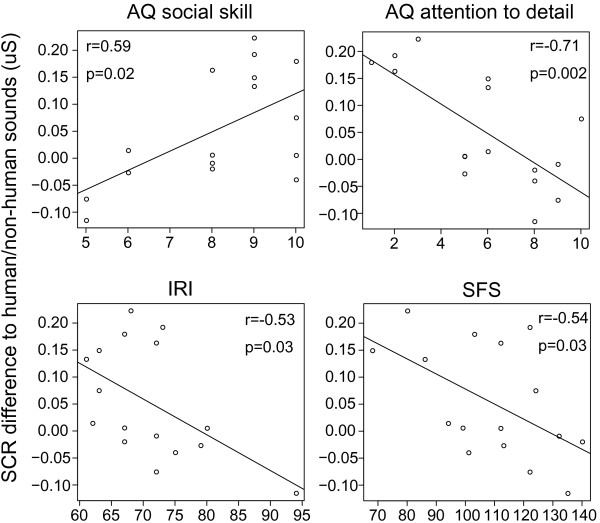
Significant correlations between the difference between the SCRs when participants heard human and non-human sounds and the scores in the social skill subscale in AQ, the attention to detail subscale in AQ, IRI, and SFS.

The lack of significant interaction among *Medication*, *Source*, and *Group* in SCRs in the five-way mixed-designed ANOVAs indicates that the NT and ASD groups had the same trend that SCRs for human sounds were larger than SCRs for non-human sounds after oxytocin inhalation. Nevertheless, the *post hoc* tests (i.e., the four two-way repeated-measurement ANOVAs) showed a greater effect of oxytocin in the ASD group than in the NT group. Based on the hypothesis that there were individual differences in the responses to oxytocin for human sounds, the Pearson correlation coefficient was computed between the difference between the SCRs for human and non-human sounds after oxytocin inhalation and participants’ age, AQ, AQ subscales, IRI, and SFS scores in the ASD group. As illustrated in Figure 3, a significant positive correlation was found between the scores in the ‘social skill’ subscale in AQ and the difference between the human and non-human sound SCRs. In addition, significant negative correlations were found between the scores in the ‘attention to detail’ subscale in AQ, IRI, and SFS and the difference between human and non-human sound SCRs. This finding supports the hypothesis that some participants, especially those with low social function and less fixated attention, were influenced by oxytocin more than others. Due to the small sample size, no post-hoc analysis could provide sufficient statistical power to investigate whether different diagnosis and medication conditions influenced their responses to oxytocin.

## Discussion

To the best of our knowledge, this is the first study to report the effect of oxytocin on the sympathetic responses (measured in terms of SCRs) toward human and non-human sounds in adults with ASD and NT adults. The present study has resulted in several interesting findings. First, after oxytocin administration, the SCRs observed in the NT group decreased. Second, after oxytocin administration, the SCRs toward human sounds became larger than the SCRs toward non-human sounds. Third, this difference in SCRs toward human and non-human sounds after oxytocin inhalation was correlated to their social function, interpersonal reactivity, and attention to detail in the ASD group.

In general, an increased SCR implies increased vigilance and/or emotional responses with a lowered neuronal threshold in sensory systems [27]. In other words, when we receive a stimulus that requires action, our brain needs to gather more information before deciding to approach or avoid that stimulus. Of all stimuli, fear is perhaps the most potent, but other stimuli that increase vigilance also increase the SCRs. Based on this interpretation, the observation that the SCRs were larger in the NT group than in the ASD group under the placebo condition indicates that, compared with individuals with ASD, the NT individuals were more vigilant or had more emotional responses to the auditory stimuli provided during the experiment. The auditory stimuli in our experiment were recorded from the natural environment. Compared with the pure tones and siren sounds used in previous studies [38-42], the auditory stimuli used in this experiment might evoke more natural responses.

While the SCRs observed after placebo inhalation became larger in the NT group than in the ASD group, the SCRs in the NT group decreased after oxytocin inhalation. Therefore, the SCRs observed in the NT and ASD groups were not significantly different after oxytocin inhalation. The reduction in the SCRs observed in the NT group might be due to attenuated amygdala activity after oxytocin treatment, which has been reported for face processing in previous studies [43-46].

In response to auditory stimuli, the brain activation patterns in the superior temporal sulcus are different for human vocal sounds and other environmental sounds [34]. However, this current study did not show any difference between autonomic responses to human and non-human sounds in the placebo condition. The lack of difference in the autonomic responses to human and non-human sounds might be due to the relatively long auditory stimuli (6 s) provided in this study compared to the short auditory stimuli used in the previous study (500 ms). Another previous study shows that a duration of 8 ms is sufficient for participants to achieve good performance in differentiating timbres and the plateau of their performance in timbre discrimination is reached when the duration is 128 ms [47]. Nevertheless, it is not clear whether the increase in stimuli duration would influence the activation in the amygdala and other brain areas that are related to the regulation of sweat glands.

Regarding the evaluation of the auditory stimuli, there are two limitations of this study. First, there was no subjective evaluation of the affective sounds during the experiment. Second, since participants were not instructed to pay attention to the auditory stimuli, their attention during the experiment was difficult to evaluate. Nevertheless, in the present study, participants were not instructed to pay attention to the auditory stimuli because a previous study found that the difference in orientation to voice between the ASD and NT participants diminished when the participants were requested to pay full attention to the auditory stimuli [48].

The difference in SCRs to human and non-human sounds after oxytocin administration was found to be negatively correlated with social function (measured with the AQ social skill subscale and SFS) and interpersonal reactivity (measured with IRI) in the ASD group. In other words, for ASD individuals, the worse their social function and interpersonal reactivity were, the larger the effect of oxytocin on differentiating human and non-human sounds. On the other hand, this difference in SCRs to human and non-human sounds was found to be negatively correlated with the ‘attend to detail’ subscale in AQ in the ASD group. This finding shows that the effect of oxytocin was stronger for the ASD participants who paid less attention to detail than for those who paid more attention to detail. In other words, the effect of oxytocin on social orientation depended on participants’ attention to the general surrounding environment.

Although oxytocin had an effect on the SCRs for human and non-human sounds, there was no such effect in relation to pleasant and unpleasant sounds observed in this study. Regarding the role of oxytocin in human affect perception, several hypotheses have been proposed: (1) oxytocin reduces social anxiety, (2) oxytocin promotes positive and pro-social behaviors, (3) oxytocin increases approach-related social behaviors while inhibiting withdrawal-related social behaviors, and (4) oxytocin facilitates social salience [49,50]. Our observation is consistent with the hypothesis that oxytocin facilitates the salience of human sounds. However, since this experiment was not designed to test these hypotheses, our observation does not rule out other hypotheses.

While this study showed an effect of oxytocin on SCRs, it did not reveal any effect of oxytocin on the amplitude changes in the PPG signals. This lack of an effect on peripheral blood vessels can be explained by its control system: peripheral blood vessels are controlled by both sympathetic and parasympathetic systems [28], while the eccrine sweat glands are entirely controlled by the sympathetic system [27]. In other words, if the parasympathetic tones changed in the same way as the sympathetic tones for human and non-human sounds after oxytocin administration, the ratio between the sympathetic tones and the parasympathetic tones would not change, and the amplitude of the PPG signals would not change.

In summary, our observation reveals that oxytocin administration changed sympathetic responses toward human and non-human sounds, especially for adults with ASD who have poorer social skills and less fixated attention. This is the first study to investigate how oxytocin inhalation changed social orientation to auditory stimuli. More work will be needed to understand the relationship between changes in SCRs induced by oxytocin administration in individuals with ASD and local changes in voice-selective brain areas influenced by oxytocin. This can be accomplished using functional imaging techniques.

## Conclusions

This study showed that a single dose of oxytocin had a short-term positive effect on orientation behaviors toward human sounds among non-human sounds. The results support the therapeutic potential of oxytocin inhalation for social orientation in adults with ASD. Future clinical trials with larger sample studies of longer duration are required if we are to fully examine the effectiveness of oxytocin as an adjunctive treatment option in the context of communication training in ASD.

## Abbreviations

AQ: Autism spectrum quotient; AS: Asperger syndrome; ASD: Autism spectrum disorders; IADS: International affective digital sounds; IRI: Interpersonal reactivity index; NT: Neurotypical; OXYR: Oxytocin receptor; PPG: Photoplethysmography; SCR: Skin conductance response; SFS: Social functioning scale.

## Competing interests

The authors declare that they have no competing interests.

## Authors’ contributions

IL conceived of the study, participated in its design, analyzed the data, and drafted the manuscript. MK participated in its design and helped to draft the manuscript. HO, TY, MT, HW, CK, TO, YT, AI, and NK helped to conduct the experiment, collect the data, and revise the manuscript. All authors read and approved the final manuscript.

## Supplementary Material

Additional file 1: Table S1 Evaluation of valence and arousal levels of the sounds used in the experiment (Bradley and Lang, 2007): mean (standard deviation) of the evaluations across subjects. Figure S1. Pre-stimulus SCLs across 29 participants for the 25 auditory stimuli presented in the experiment in the oxytocin session and the placebo session. The *error bars* are standard errors. Click here for file
