# Supplementary material for: The effect of intranasal oxytocin versus placebo treatment on the autonomic responses to human sounds in autism: a single-blind, randomized, placebo-controlled, crossover design study
Source: Mol Autism. 2014 Feb 28;5:20. doi: 10.1186/2040-2392-5-20 (PMC4015306; doi:10.1186/2040-2392-5-20)
Supplement: Additional file 1: Table S1 — Evaluation of valence and arousal levels of the sounds used in the experiment (Bradley and Lang, 2007): mean (standard deviation) of the evaluations across subjects. Figure S1. Pre-stimulus SCLs across 29 participants for the 25 auditory stimuli presented in the experiment in the oxytocin session and the placebo session. The error bars are standard errors. [file 2040-2392-5-20-S1.docx]

**Additional file**

**Table S1**

Evaluation of valence and arousal levels of the sounds used in the experiment (Bradley and Lang, 2007): mean (standard deviation) of the evaluations across subjects.

|  |  | pleasure | arousal |  | pleasure | arousal |
| --- | --- | --- | --- | --- | --- | --- |
| First sound | Beer-open | 6.82±1.60 | 4.51±1.60 |  |  |  |
| Pleasant human | Native song | 6.17±1.99 | 5.29±1.99 | Baby laughing | 7.64±2.10 | 6.03±2.10 |
|  | Music box | 6.01±2.19 | 5.65±2.19 | Sports bar | 7.65±1.58 | 7.12±1.58 |
| Pleasant non-human | bagpipe | 6.21±2.12 | 5.07±2.12 | Colonial music | 6.53±1.66 | 5.84±1.66 |
|  | Guitar | 6.98±1.90 | 5.23±1.90 | Rock & roll | 7.90±1.53 | 6.85±1.53 |
| Unpleasant human | sneeze | 3.86±1.70 | 5.19±1.70 | Baby cry | 2.75±1.68 | 6.51±1.68 |
|  | wheeze | 2.44±1.34 | 6.31±1.34 | Man scream | 1.99±1.41 | 7.28±1.41 |
| Unpleasant non-human | Engine failure | 3.15±2.01 | 6.32±2.01 | Rattle snake | 3.55±1.99 | 6.98±1.99 |
|  | Animal growl | 3.37±1.64 | 6.39±1.64 | Bees | 2.16±1.33 | 7.03±1.33 |
| Pleasant non-arousal | Harp | 7.44±1.41 | 3.36±1.41 | choir | 6.90±1.69 | 3.43±1.69 |
|  | Country night | 5.59±1.79 | 3.71±1.79 | yawn | 5.26±1.58 | 2.88±1.58 |

**Figure S1**

Pre-stimulus SCLs across 29 participants for the 25 auditory stimuli presented in the experiment in the oxytocin session and the placebo session. The error bars are standard errors.
